# Automatic cardiac evaluations using a deep video object segmentation network

**DOI:** 10.1186/s13244-022-01212-9

**Published:** 2022-04-08

**Authors:** Nasim Sirjani, Shakiba Moradi, Mostafa Ghelich Oghli, Ali Hosseinsabet, Azin Alizadehasl, Mona Yadollahi, Isaac Shiri, Ali Shabanzadeh

**Affiliations:** 1 Research and Development Department, Med Fanavarn Plus Co., 10th St. Shahid Babaee Blvd., Payam Special Zone, 3187411213 Karaj, Iran; 2 Department of Cardiovascular Sciences, KU Leuven, Leuven, Belgium; 3 Cardiology Department, Tehran Heart Center, Tehran University of Medical Sciences, Tehran, I.R. Iran; 4 Echocardiography and Cardiogenetic Research Centers, Cardio-Oncology Department, Rajaie Cardiovascular Medical and Research Center, Tehran, Iran; 5 Division of Nuclear Medicine and Molecular Imaging, Geneva University Hospital, 1211 Geneva 4, Switzerland

**Keywords:** LV measurements, RV measurements, Segmentation, Deep learning, Convolutional neural network

## Abstract

**Background:**

Accurate cardiac volume and function assessment have valuable and significant diagnostic implications for patients suffering from ventricular dysfunction and cardiovascular disease. This study has focused on finding a reliable assistant to help physicians have more reliable and accurate cardiac measurements using a deep neural network. EchoRCNN is a semi-automated neural network for echocardiography sequence segmentation using a combination of mask region-based convolutional neural network image segmentation structure with reference-guided mask propagation video object segmentation network.

**Results:**

The proposed method accurately segments the left and right ventricle regions in four-chamber view echocardiography series with a dice similarity coefficient of 94.03% and 94.97%, respectively. Further post-processing procedures on the segmented left and right ventricle regions resulted in a mean absolute error of 3.13% and 2.03% for ejection fraction and fractional area change parameters, respectively.

**Conclusion:**

This study has achieved excellent performance on the left and right ventricle segmentation, leading to more accurate estimations of vital cardiac parameters such as ejection fraction and fractional area change parameters in the left and right ventricle functionalities, respectively. The results represent that our method can predict an assured, accurate, and reliable cardiac function diagnosis in clinical screenings.

## Key points

- EchoRCNN is a deep video object segmentation neural network.
- EchoRCNN is based on the Mask Region-Based Convolutional Neural Network (Mask R-CNN) image segmentation architecture.
- This network is trained on the ultrasound cardiac series to delineate ventricles.
- Ejection Fraction for the left ventricle is estimated from the network output.
- The output area can determine Fractional Area Changes for the right ventricle.

## Introduction

Quantification of the Left Ventricle (LV) and Right Ventricle (RV) function on echocardiography series in terms of characterizing the ventricular size, volume, and Ejection Fraction (EF) is a key step for cardiac disease diagnosis and treatment [1]. By the American Society of Echocardiography efforts, these parameters account for the standard clinical measurements in guidelines [2]. This importance comes from the convenience and availability of echocardiography, making it the most frequently utilized imaging system in clinical routines.

LV and RV manual segmentation for functional evaluation is highly time-consuming and prone to inter- and intra-expert variability. This issue intensifies since a human observer looks at the entire cardiac cycle in clinical routine to find end-systolic and end-diastolic frames. Despite many achievements to overcome these issues by automating the process, the problem is still challenging due to low signal to noise ratio, edge dropout, low contrast, the presence of shadows produced by dense muscles, and a considerable amount of processing data in case of entire cardiac cycle segmentation [3].

Most of the studies in this era focus on LV or RV segmentation in specific frames [4–16]. However, due to valuable information in a cardiac cycle sequence, it is highly effective to utilize this information for cardiac chamber segmentation. The most remarkable studies in video object segmentation task have contributed to the Densely Annotated Video Segmentation (DAVIS) challenge [17], an annual competition in this field. MaskRNN [18], which is a representative of algorithms that use optical flow information to have insights into movements, and Reference-Guided Mask Propagation (RGMP) [19], which contains a reference stream along its main path, are successful examples of semi-supervised architecture on the DAVIS dataset. There are a few studies in the context of echocardiography series segmentation. Researchers in [20] targeted the cardiac sequence segmentation using their previously presented U-net version [9] to segment LV in each frame in echocardiography sequences. In another work [3], Deep Belief Network (DBN) and dynamic models for LV tracking are combined based on the Sampling Importance Resampling (SIR) [21]. Their proposed model takes advantage of using features extracted from all previous and current frames and previous segmentation contours. In [22], a method with two network streams is proposed. The first one is the spatiotemporal stream which takes the input sequence and estimates the EF parameter, and the second one processes each frame independently to segment the desired area. The network is semi-supervised and takes the weak manual segmentation of the end-diastolic and end-systolic frames as inputs [22].

This study proposes a novel network architecture, EchoRCNN, specifically for echocardiography series. We consider training this network for two separate LV and RV segmentation tasks in echocardiography sequences as a semi-supervised procedure because it is essential to involve medical expert control on the algorithm predictions. The network combines Mask Region-Based Convolutional Neural Network (Mask R-CNN) structure [23], which is a robust image segmentation neural network based on Faster R-CNN object detection network, with RGMP video object segmentation network [19]. It consists of two streams, one takes the original frame and the previous mask, and the other takes the reference frame as inputs inspired by the RGMP network [19]. The outputs of these two streams are then concatenated as the input of the last stage, and the network's output is a binary mask for each frame. Then, using post-processing methods on the segmented area leads to estimating particular echocardiography parameters. This approach helps radiologists trace heart functionality through time at a reasonable speed.

## Materials and methods

**Fig. 1:**
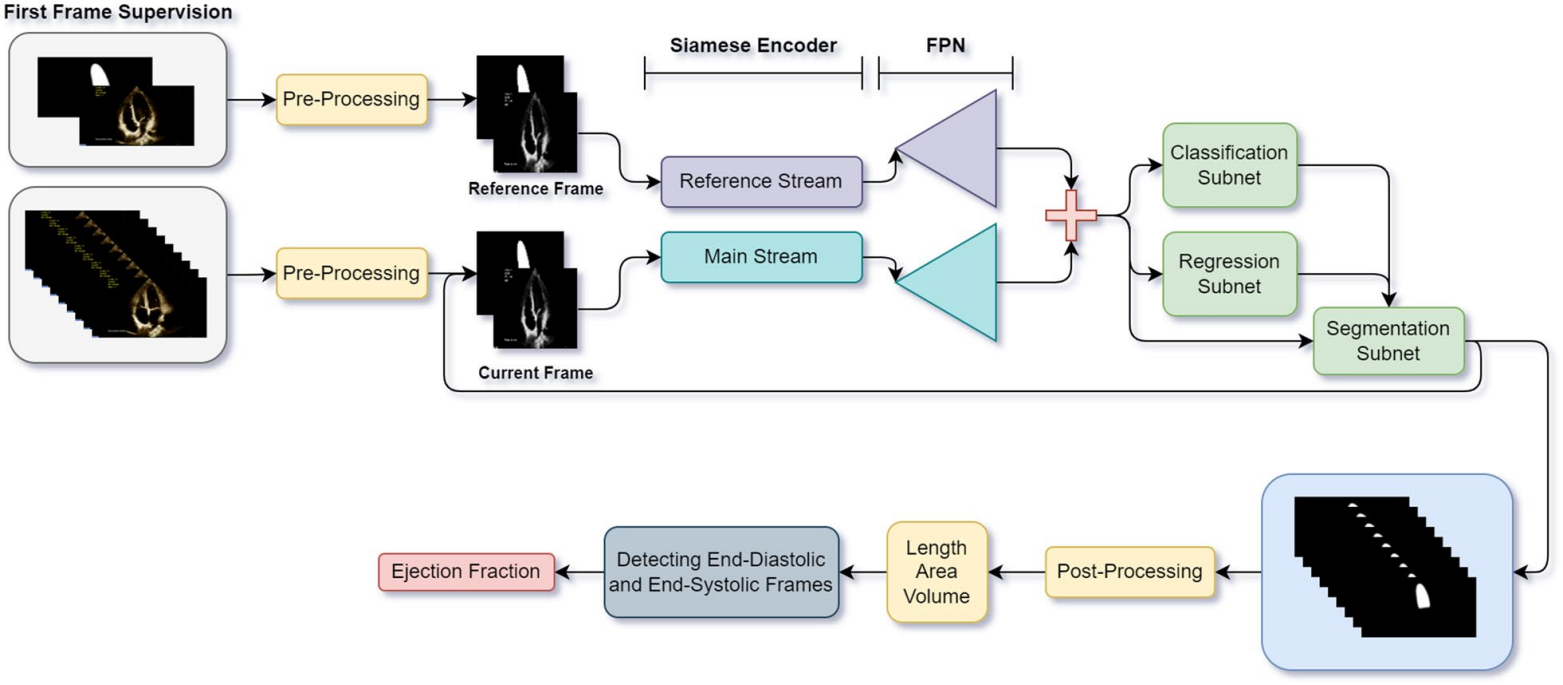
The workflow of the algorithm for LV series. After pre-processing, the first frame and its supervision enter into the reference stream, and the following frames enter into the main stream one after the other after pre-processing. The inputs are passed through the Siamese encoder, FPN, and three final subnets (classification, regression, and segmentation subnets), which are the main components of EchoRCNN. Then, the main parameters of LV functionality (length, area, and volume) are extracted after post-processing on the predicted masks. By having these parameters for each frame, end-diastolic and end-systolic frames are detected, and finally, the EF parameter can be calculated.

This section describes the whole process of the ventricles' assessments from gathering data to extracting parameters from the segmented series. The process of the LV calculations is illustrated in Fig. 1.

### Description of the left ventricle dataset

A collection of 2D echocardiography series was prepared. The videos were acquired from Tehran Heart Center, Tehran, Iran, from 2017 to 2019. The dataset was gathered by Affiniti 70 ultrasound imaging system (Philips). The whole data consist of more than 3000 videos obtained from different patients in different view angles. Three experts investigated all videos and selected 750 four-chamber view series with proper LV shapes. Each selected video has 45 frames on average. Three experts delineated the LV region in each frame using the Qlab Cardiac Analysis (cardiovascular ultrasound quantification software from Philips Co.).

Delineation was performed using Auto 2D Quantification (a2DQ) tool in the Qlab Cardiac Analysis. Given weak supervision of LV borders in the end-diastolic frame by a2DQ, users manually refine the points on walls to correct the estimated region for LV. The software automatically segments all frames according to the end-diastolic delineation, and finally, the user refines the segmented region by modifying the borders in the end-systolic frame. The estimated LV volume at end-diastolic and end-systolic frames, the estimated EF, and the end-diastolic and end-systolic frame numbers were also extracted from the a2DQ tool for further evaluations and analysis.

The resolution of frames is $1110\times 581$. The dataset was split into train and test sets, such that we have 675 videos in the training dataset and 75 videos in the test dataset.

### Description of the right ventricle dataset

A set of 2D echocardiography series was prepared and acquired from Rajaie Cardiovascular Medical and Research Center, Tehran, Iran. The Echocardiography series were gathered by EPIQ 7 imaging system (Philips). Experts have selected 80 patients for data acquisition. At most, three echocardiography series in four-chamber view were obtained with up to 60 frames from each patient. Finally, 175 series were extracted from the whole gathered dataset. Two different experts delineated the RV region in each frame using TOMTEC Imaging Systems GmbH separately.

Delineation was performed using an RV segmentation tool in the mentioned software. The tool creates weak supervision of RV walls in the end-diastolic phase. After the user modifies the borders to have the correct segment of the RV region in the end-diastolic frame, the software will automatically segment other frames. Finally, the user can correct the RV region's border points in the end-systolic frame to segment the sequence appropriately.

The resolution of frames is $800\times 600$. The dataset was split into train and test sets, such that we have 160 videos in the training dataset and 15 videos in the test dataset.

### Pre-processing

Both LV and RV video frames are converted to grayscale images and resized to $128\times 256$. For each image, min–max scaler normalization is performed, and each image's pixels are normalized to be in the range of 0 and 1. The ground truth masks are also binarized to have only 0 and 1 values.

We have a two-channel input for each batch of data for the main stream containing the current grayscale image and the previously predicted mask. A two-channel input for the reference stream includes the reference grayscale image and the ground truth mask for the reference image.

### EchoRCNN architecture

**Fig. 2:**
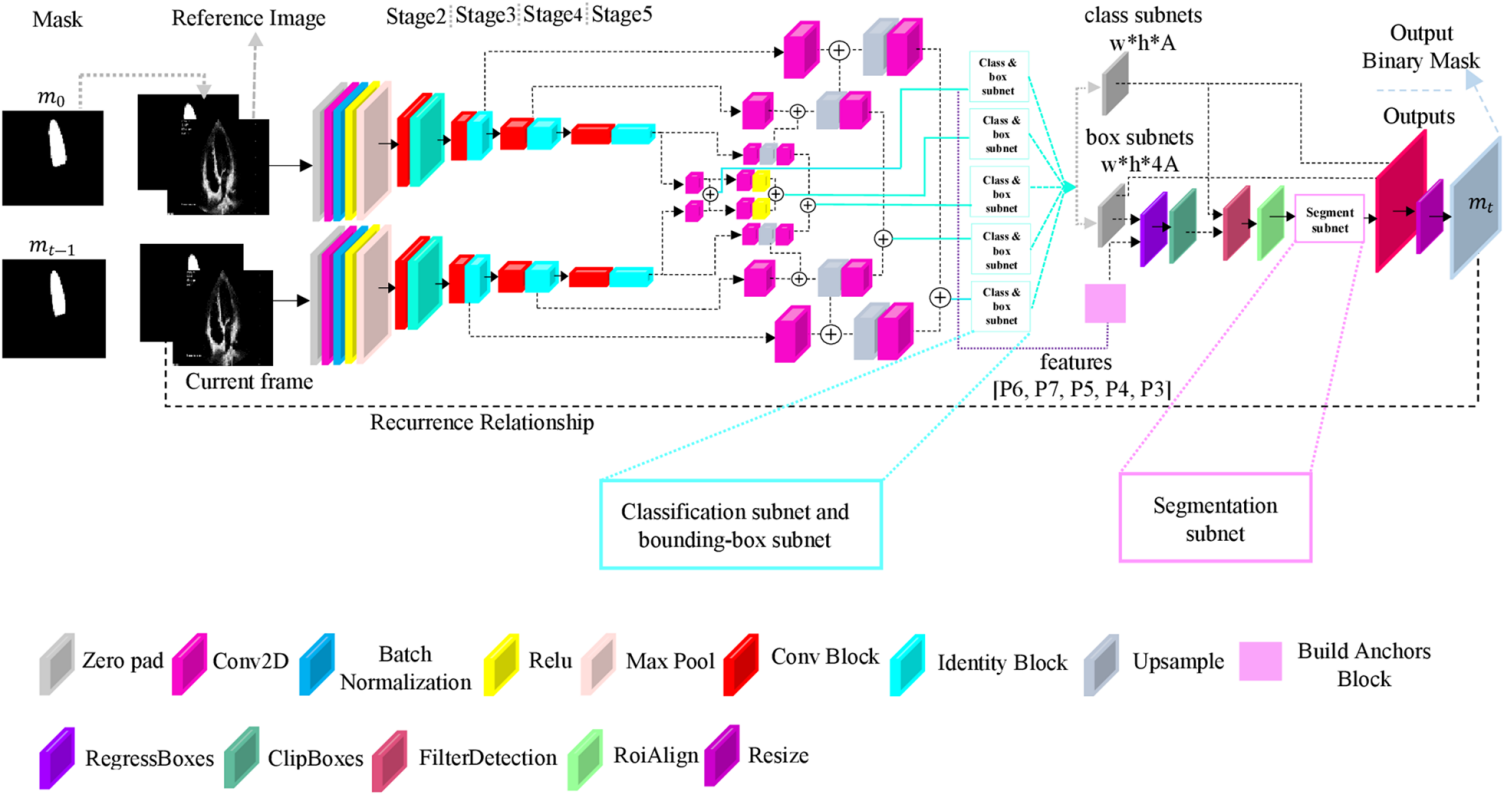
The complete architecture of EchoRCNN

The network architecture is inspired by RGMP, RetinaNet, and Mask R-CNN networks introduced in [19, 23, 24]. It consists of a Siamese encoder followed by a Feature Pyramid Network (FPN) [25], a classification subnet, a regression subnet, and a segmentation subnet. Figure 2 shows the complete architecture of the video segmentation network. The Siamese encoder path includes the main stream and the reference stream. The main stream takes the current original frame and the previous frame's predicted mask (guidance mask). The one-channel image and one-channel guidance mask are concatenated to make a two-channel image. A reference frame, the first frame of the cardiac cycle, is fed into the reference stream. The user must annotate this frame. Consequently, the reference stream takes the original frame in grayscale mode concatenated with its annotation as an input. Both streams are based on ResNet50 [26], a five-layer feature extraction architecture with residual blocks.

The outputs of stage 3, stage 4, and stage 5 in both streams are then fed into the FPN for each stream. Same as [24], we have used FPN to produce five feature pyramids P3, P4, P5, P6, and P7. All of the feature maps in this part of the network have 256 channels, and each level is used for object detection on a different scale. Each of the produced feature pyramids is fed into both classification and regression subnets. The classification subnet's prediction is the probability of an object's attendance at each spatial position for A anchors (A is the number of anchors). This subnet is a small, fully convolutional network introduced in [24] that takes each feature map with 256 channels from pyramid levels. It consists of four convolutional layers (with 3 × 3 kernels and 256) followed by ReLU activations, a convolutional layer with A filters, and a sigmoid activation layer. The regression subnet is like the classification subnet with nuance differences in the last layer. The last layer is a convolutional layer with 4A filters. This network regresses the offset from each anchor box to a close ground truth object if it exists. These four outputs predict the anchor's and the ground truth box's affiliate offset for each A anchor per spatial location.

On the other hand, the feature pyramids are fed into the anchor building block, producing the translation-invariant anchor boxes resemblance to [24]. 9 anchors are used at each pyramid level with three different aspect ratios and scales with 322 to 5122 areas on P3 to P7 pyramid levels. Each anchor is assigned to a classification target and a vector of box regression targets. Using Intersection-over-Union (IoU) metric, anchors are allocated to the ground truth object boxes by the threshold of 0.5, and they are considered background if their IoU is in [0, 0.4). Otherwise, the anchor is unassigned, which means the IoU is in [0.4, 0.5), and these anchors are ignored during the training procedure. Box regression targets are obtained by calculating the offset between each anchor and its assigned object box; conversely, an anchor is omitted if there is no assignment.

**Fig. 3:**
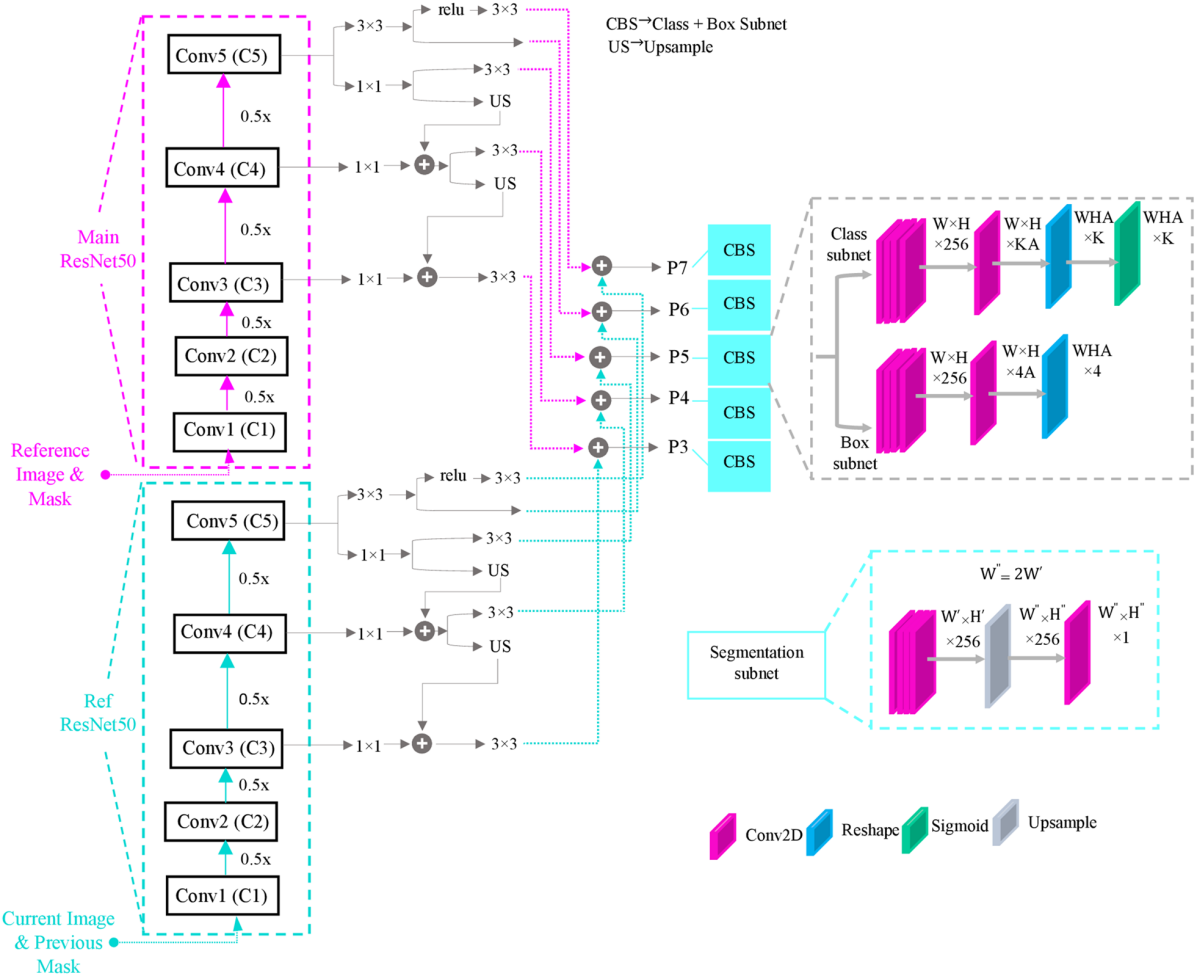
Details of segmentation and box subnets in the EchoRCNN architecture (inspired from RetinaNet)

Regression subnets output and the anchors produced by the anchor building block are fed into the Regress-Boxes layer to apply regression to the anchors. To fix the boxes' size to be inside the image shape, they are passed through the ClipBoxes layer. New boxes with the classification subnet output are fed into the Filter detection layer in which the top predictions from all levels are elided, and by exerting the non-maximum suppression and a threshold of 0.5, the final predictions are generated. The final boxes are fed into the RoiAlign layer to fix the misalignment and produce suitable inputs for the segmentation subnet. The segmentation subnet is a simple fully convolutional network with four convolutional layers (with 3 × 3 kernels and 256), followed by an upsample layer, two convolutional layers, and a sigmoid activation layer. Based on an index of the highest score of object presence probability, the binary mask output is chosen, resized, and feedback to the input as the previous mask. Figure 3 indicates details about subnets.

### Training the network

Two instances of the network illustrated in Fig. 2 are trained end-to-end using the backpropagation algorithm on LV and RV datasets separately. The initial weights for the ResNet50 backbone in the main stream and the reference stream in the LV segmentation network are ImageNet weights, and the rest of the layers have random initial weights. For RV segmentation, the initial weights of the ResNet50 backbone are obtained from the trained LV segmentation model to handle the data shortage of the RV segmentation task. The other layers in the RV segmentation network have random initial weights. The sequences are randomly selected from the whole training data. The first frame is fed into the reference stream, and the rest are fed into the main stream one by one as a batch. There is no fine-tuning in the inference procedure because of the time limitation that exists in the task of echocardiography examinations.

The loss function used in the training procedure consists of 3 losses, including regression loss, classification loss, and segmentation loss. We have used the Focal loss for classification loss suggested by [27], a loss function used in imbalanced dataset cases. In the video object segmentation task, especially in the proposed study (a single instance segmentation), most of the pixels belong to the background class, so the assigned category for most anchors should be set to zero or background class. Therefore, the classification branch is somehow imbalanced. Weighted loss functions are defined to penalize the network more when it wants to put an anchor in the majority class to address this problem. Equation (1) shows the formula of the Focal from [27].

1 $${\text{FL}}(p_{t} ) = - \alpha_{t} (1 - p_{t} )^{\gamma } \log (p_{t} )$$

We have used the smooth-L1 function proposed in [28] for the regression loss. Equation (2) shows the formula of this loss function. The function's key component is the σ hyperparameter, which divides the positive section into two parts. For the targets between 0 and *σ*, the function acts the same as the L2 loss function. For the targets beyond the σ parameter, the function is the same as the L1 loss function. This structure helps to avoid over penalizing the outliers.

2 $$F(x) = \left\{ {\begin{array}{*{20}l} {0.5\frac{{x^{2} }}{\sigma }} \hfill & {{\text{if}}\;|x| < \sigma } \hfill \\ {|x| - 0.5\sigma } \hfill & {\text{o.w.}} \hfill \\ \end{array} } \right.$$

We have used the average binary cross-entropy in the segmentation branch as the loss function.

### Finding the left ventricle parameters

After training the network and predicting the LV region, we will go deep into this task's primary purpose, which is estimating the cardiac most valuable parameters derived from the echocardiography sequence. The LV's most important parameters are length, area, volume, and EF. EF is defined as the amount of blood LV pumps out in each heart contraction. Theoretically, it is the stroke volume ratio (the difference between the LV end-diastolic and end-systolic volumes) to the LV end-diastolic volume. As these significant parameters are defined in the diastolic or systolic frames, the first step is to detect them between the whole sequence frames.

According to [29], the end-diastolic frame is determined using some clues like mitral valve closure, R-wave of Electrocardiogram (ECG), and the LV volume's maximum size. There are also mitral valve opening, the minimum size of the LV volume, aortic valve closure, and T-wave signs for detecting the end-systolic frame. As we have all the frames' segmented area for LV and it is possible to estimate the LV volume from this segmented surface, the most proper way to detect end-diastolic and end-systolic frames is to use minimum and maximum volume clues. Our previous work [8] introduced a post-processing method to calculate LV length, area, and volume from the segmented part in each frame by finding three critical points on LV shape. These points, placed on the apex and the mitral valve joints, are detected from the segmented part's convex hull.

Further calculations from the distances between these points can lead us to the LV length, and by having the number of pixels inside the segmented part, the LV area can be estimated. Using modified Simpson's Rule [30], by having the length and area of the LV in the 2D plane, the volume can be determined by Eq. (3), where *L* and *S* denote LV length and area, respectively.

3 $$V = \frac{{8S^{2} }}{3\pi L}$$

Here, we have used this method to estimate LV volume in each frame and perform the following steps to assess LV parameters.

1. Estimate LV area, length, and volume in each frame
2. Find the frame where the LV volume is maximum; this frame is the end-diastolic frame
3. Find the frame where the LV volume is minimum; this frame is the end-systolic frame
4. Calculate EF

### Finding the right ventricle parameters

After a successful training procedure, we should continue the vital quantifications in RV functionality. For this chamber, the essential parameters are area and Fractional Area Change (FAC), which is the percentage of the area change within the RV region between end-diastolic and end-systolic frames [29].

As mentioned in the previous section, the first step is to estimate which frames are in end-diastolic and end-systolic phases. According to [30], biological signs of end-diastolic frames are not involved in RV functionality; therefore, RV segmentation and area tracing cannot define the exact end-diastolic and end-systolic frames, so a supervisor must determine them. Thus, the following steps are performed to estimate the FAC parameter.

1. Tracking the RV area through the time
2. Define end-diastolic and end-systolic frames in the whole sequence by an expert.
3. Extract the area of the RV region in the selected frames
4. Calculate the FAC parameter for RV

### Implementation details

We have used a system with 16 GB of RAM, a GPU-based graphic card with 2176 CUDA cores (GeForce RTX 2060-A8G), and an Intel Xeon CPU. The network was implemented in Python environment with Tensorflow 2 [31] and Keras 2.2.4 [32]. The hyperparameter optimization is done by a random search for learning rates and regularization parameters on a few iterations. We have used Stochastic Gradient Descent (SGD) as an optimizer to train the network, and we have chosen the learning rate to be 10e-5.

### Evaluation metrics

The method is evaluated in two main steps: segmentation procedure and parameter estimation. For the first step, which is upon the network prediction, the segmentation task's robustness is evaluated based on some of the key metrics in this field, such as Dice Similarity Coefficient (DSC), Jaccard Similarity Coefficient (JSC), precision, and recall. In the second step, LV and RV parameters extracted from the segmented frames using post-processing methods are compared to the ground truth parameters. Root Mean Squared Error (RMSE), Mean Absolute Error (MAE), R-squared factor, and Cronbach's α are the metrics used for this part. There is a thorough explanation of each metric in the “Appendix” section.

## Results

Table 1 shows the segmentation results for LV and RV regions in EchoRCNN compared to the MaskRNN and RGMP networks. As mentioned before, we have used DSC, JSC, precision, recall, and F1 score to evaluate the method. In terms of these metrics, the results illustrate robustness and reliability. LV video object segmentation has been assessed on 75 echo series. The number of test sequences for RV is 15 since the whole of videos for RV was significantly less than LV videos. EchoRCNN has reached 94.03% of DSC and 88.83% of JSC for LV and 94.97% of DSC, and 90.61% of JSC for RV segmentation tasks. The outperformance of the EchoRCNN compare to the MaskRNN and RGMP is evident in Table 1.

**Table 1:** Final segmentation results of LV and RV test sequences, comparison between the proposed network EchoRCNN and the MaskRNN and RGMP architectures

Network	Region	DSC^a^	JSC^b^	Precision	Recall
EchoRCNN	Left ventricle	94.03 ± 0.01	88.83 ± 0.03	94.85 ± 0.02	93.37 ± 0.02
	Right ventricle	94.97 ± 0.02	90.61 ± 0.04	97.03 ± 0.01	93.14 ± 0.04
MaskRNN	Left ventricle	85.04 ± 0.01	70.56 ± 0.02	83.31 ± 0.01	90.99 ± 0.006
	Right ventricle	69.25 ± 0.27	59.68 ± 0.3	80.78 ± 0.21	74.23 ± 0.32
RGMP	Left ventricle	81.96 ± 0.1	70.97 ± 0.12	81.02 ± 0.16	74.55 ± 0.14
	Right ventricle	65.41 ± 0.26	55.26 ± 0.25	75.76 ± 0.15	63.03 ± 0.3

^a^Dice similarity coefficient (DSC)

^b^Jaccard similarity coefficient (JSC)

After obtaining the segmented RV and LV parts, their vital parameters are estimated through the post-processing methods explained before. The LV parameters include length, area, volume, and EF, and the RV parameters consist of the area and FAC. We have reached 0.72 R-squared for the EF parameter in LV and 0.89 for the FAC parameter in the RV series. Cronbach's α has been used for measuring internal consistency between the prediction values and the corresponding ground truth. The internal consistency for the EF parameter in LV has been reached 0.92 and 0.97 for the FAC parameter in RV. Tables 2 and 3 illustrate regression metrics for each of these parameters in both end-diastolic and end-systolic phases for LV and RV separately. RGMP and MaskRNN networks have weak predictions for end-systolic phases, especially in RV segmentation. In other words, the error is propagating through time, and when the chamber dwindles, the network cannot track the borders prosperously. In our investigations, these two networks cannot segment any region in the end-systolic phase in most of the sequences. Therefore, further calculations of volume, area, and EF are not reliable.

**Table 2:** Final estimation results of LV parameters, length, area, volume and EF which is calculated directly from the LV volume in end-systolic and end-diastolic phases

Parameter	Phase	RMSE	MAE	*R*^2^	Cronbach’s α
Length	ED	0.26	0.20	0.82	0.95
	ES	0.29	0.23	0.83	0.96
Area	ED	1.34	1.09	0.89	0.97
	ES	1.18	0.99	0.89	0.97
Volume	ED	8.13	6.37	0.87	0.96
	ES	5.33	4.42	0.86	0.96
EF		3.91	3.13	0.72	0.92

**Table 3:** Final estimation results of RV parameters, area and FAC which is directly calculated from the RV area in end-systolic and end-diastolic phases

Parameter	Phase	RMSE	MAE	*R*^2^	Cronbach’s α
Area	ED	1.80	1.06	0.97	0.99
	ES	0.81	0.64	0.96	0.99
FAC		5.75	2.03	0.89	0.97

**Fig. 4:**
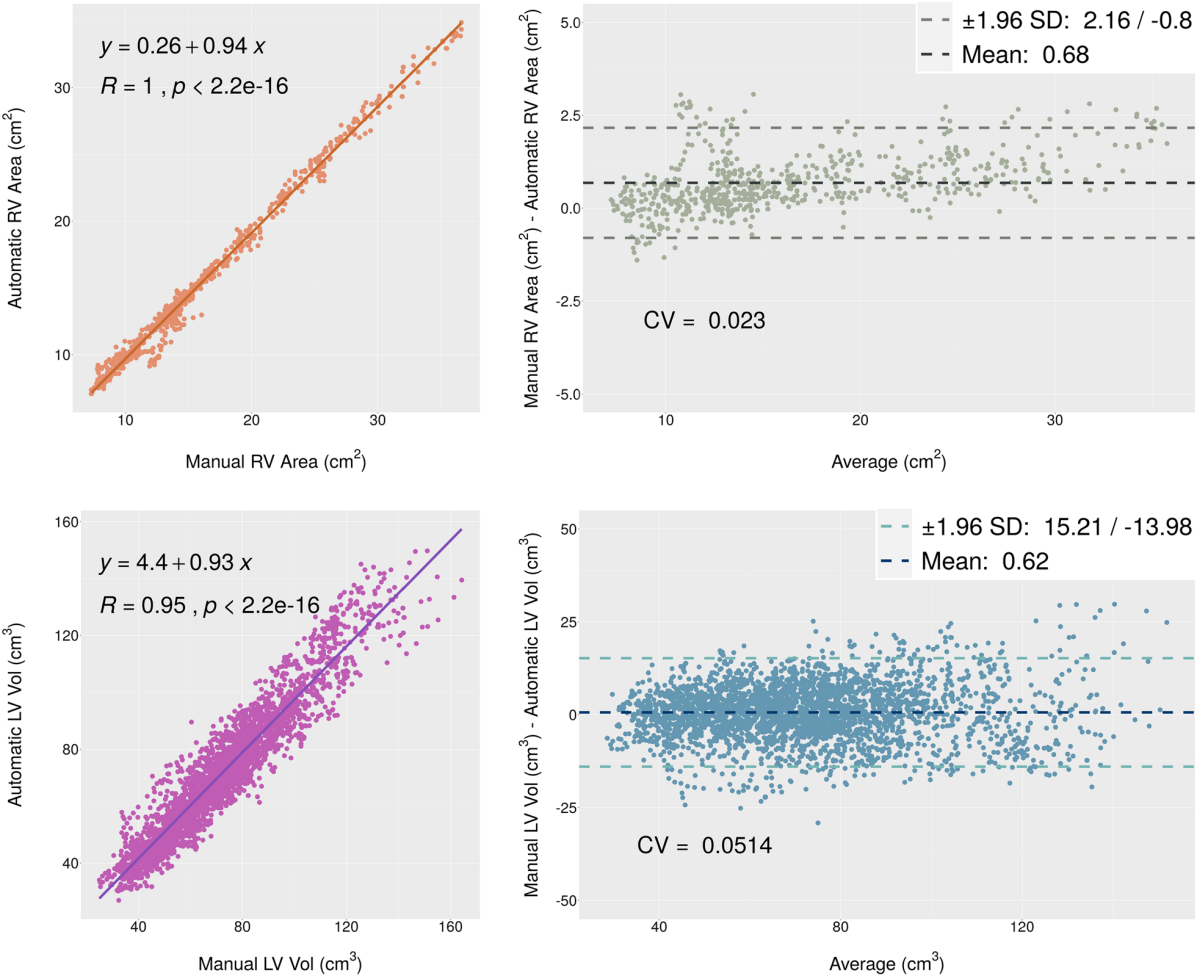
The Bland and Altman and correlation diagrams for the LV volume and RV area which are key parameters in calculating EF and FAC

The Bland and Altman analysis and Pearson test are also performed on the LV volume and the RV area through the whole dataset frames. Figure 4 indicates the plots of these two analyses. For the LV volume and RV area, a correlation of 0.94 and 0.93 are achieved, respectively. Therefore, a strong correlation exists between the manual and predicted parameters.

We can better visualize the differences between manual and predicted values for each parameter from the Bland and Altman diagrams. The width of confidence intervals for LV length, area, and volume were 0.90 cm, 5.27 cm^2^, and 29.19 cm^3^, and the mean values were 0.12 cm, 0.46 cm^2^, and 0.62 cm^3^, respectively. The width of the confidence interval for the RV area is equal to 2.96 cm^2^, with a mean value of 0.68 cm^2^.

**Fig. 5:**
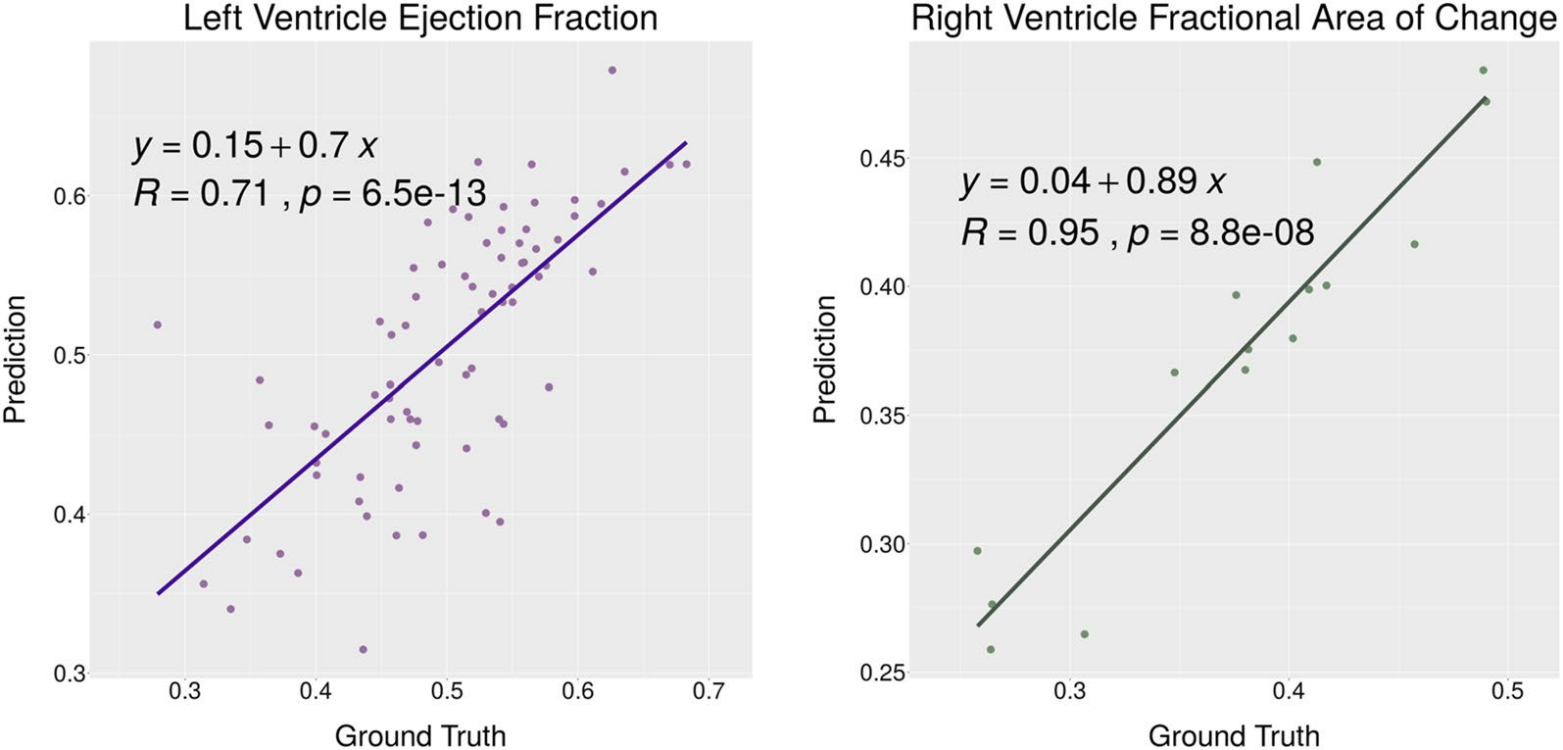
Correlation diagrams for the EF and FAC

The correlation test is used to evaluate two vital parameters EF from LV functionality and FAC from RV functionality. Figure 5 indicates that the correlation between manual and automatic parameters is thoroughly acceptable.

**Fig. 6:**
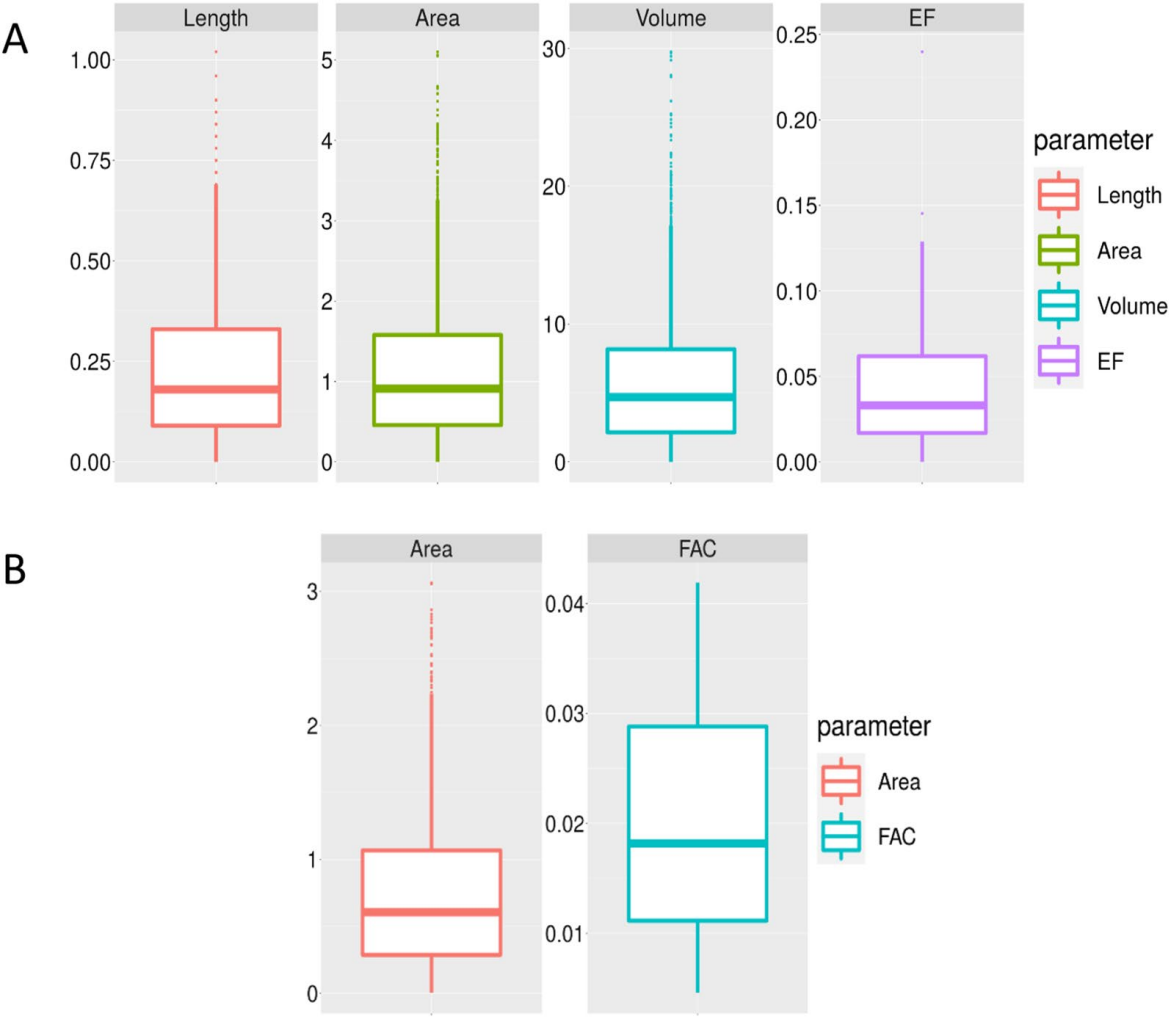
The box plot of the parameters. **A** Length, area, volume and EF for LV and (**B**) area and FAC for RV, the diagram show the quantiles of error between the true values and the predicted ones

Figure 6 is the boxplots of the error between each parameter's actual and estimated values. This plot shows the quantiles of the absolute error for LV parameters, length, area, volume, and EF, and RV parameters, area, and FAC. It can be observed that the boxes are dense, and specifically for the EF and FAC parameters, there are no annoying outliers.

**Fig. 7:**
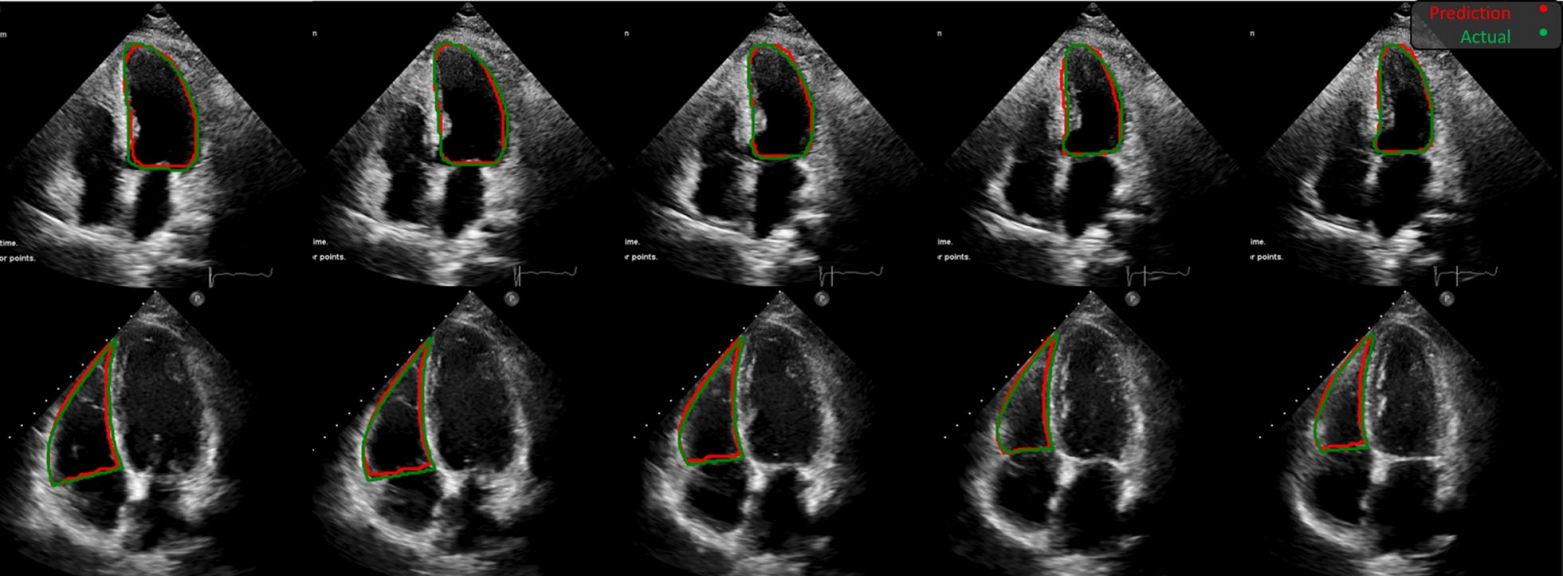
The comparison between the predicted and the ground truth regions in five frames sampled from two sequences in the LV and RV test series. The green borders indicate actual regions, and the red ones are the EchoRCNN predicted borders

In Fig. 7, five frames sampled from two selected LV and RV test sequences are depicted with actual and predicted borders between end-diastolic and end-systolic phases. Obviously, the walls are well followed in the predicted regions.

## Discussion

EchoRCNN is a novel video object segmentation network based on the robust image segmentation architectures, Mask R-CNN and RetinaNet, and RGMP video object segmentation network, specifically designed to extract cardiac features from echocardiography series in four-chamber view. The method is semi-supervised as it takes the manual segmentation of the first frame of each sequence as a guide. Using the segmentation results of this network and the following post-processing algorithms leads to accurate estimations of LV and RV functionality factors. EF and FAC, which are vital measurements in clinical screenings, are two main objectives of this research. For calculating these two, the LV length, area and volume, and the RV area are also estimated in end-diastolic and end-systolic phases. There is a clinical definition for finding end-diastolic and end-systolic frames based on the changes in LV volume through the cardiac sequence; therefore, the method can automatically find these phases by following the segmented part through the echocardiography series.

EchoRCNN's key feature is the existence of two streams: the main and the reference streams based on the concepts in the RGMP network and having a recurrent path to handle the sequencing data. The main stream accepts the current frame and the previously predicted mask, and the reference stream gets the reference frame which is assumed to be the first frame and its ground truth mask as inputs. A recurrent neural network component connects predictions over time in a unifying framework and benefits the time dependencies between video frames. Both streams include layers for feature extraction. Based on the Mask R-CNN network, the rest of the network contains three branches for segmentation, classification, and bounding box regression tasks. The observation on both datasets for the RV and LV segmentation indicates this network's strength and accuracy in the pixel classification task.

Compared to some popular video object segmentation networks such as MaskRNN, and RGMP, which have excellent performance in the DAVIS challenge, the proposed EchoRCNN outperforms other methods in the echocardiography series. Some assumptions make the network prediction more straightforward compared to the DAVIS challenge. One of the advantages of the cardiac dataset over the natural video datasets like DAVIS is the invariant property of echocardiography videos. Therefore, the test examples have the same structure as the training set, and it seems there is no need to fine-tune.

There are also some concerns in the ventricle chambers' video segmentation. First is the inference speed, which matters because the algorithm should respond during the patient's examination. Second is the intrinsic noise of ultrasound images, making it hard for the model to perceive image sequences. EchoRCNN is a learning approach attempts to use echocardiography sequences' advantages and handle the mentioned issues.

Some methods, such as the One-Shot Video Object Segmentation (OSVOS) network [27], lack interest in frames' correlation through time, even on the DAVIS dataset. In other words, the network with similar architectures does not have any recurrent path and cannot relate the movements between two consecutive frames. MaskRNN has used the optical flow algorithm to add information about the different movements from one frame to another to tackle this problem. It also has a recurrent path to have a better insight into the predictions for previous frames. The most crucial disadvantage of this network is time and calculation costs. The optical flow algorithm is an expensive and time-consuming method. The RGMP network removed the optical flow and created a novel architecture using a reference stream and choosing a reference frame that makes the network retain the proposed object's general structure. Despite these networks' remarkable results in the DAVIS challenge, their performance on the echocardiography dataset is not satisfying due to the intrinsic differences between the DAVIS dataset and echocardiography series. As observed in Table 1 and explained in the result section, the performance of these networks decreases in the last frames of the cycle. When the cardiac cycle reaches the end-systolic phase, as the contraction happens and the papillary muscles appear, the network becomes more unreliable in prediction, and the error propagates through the following frames. For some of the sequences, the RGMP and MaskRNN cannot predict any regions in the end-systolic phase. EchoRCNN has a robust architecture that can remarkably follow the chamber through frames even when contraction occurs in the end-systolic phase. Using Mask R-CNN base components and the idea of having a reference region for prediction on each frame inspired from RGMP makes the network robust to error propagation through the time in noisy echocardiography series.

The proper segmentation procedure makes the following post-processing methods for extracting the cardiac parameters more reliable. An ellipsoid model with single-plane data, which assumes that the chamber is symmetric about the longitudinal axis, is used to extract LV volume and EF parameters. The algorithm has been proved to be fast and reliable [33, 34]. According to recommendations from the American Society of Echocardiography [35], it is more accurate to use an ellipsoid model with biplane data to calculate LV volume, meaning that two views are required to assess this parameter. Nonetheless, processing an extra echocardiography series (the second view) for each patient might double calculations, which means the algorithm will slow down. Therefore, a slight gain in the accuracy should not be compromised by the loss of speed, so it has been chosen to use a single view and a single-plane model to keep the algorithm fast enough to be a useful assistant for radiologists.

It is shown that there is an acceptable inconsistency in two main parameters, EF and FAC, extracted from LV and RV functionalities, respectively. By comparing our results with the manual measurements and considering the difference between predicted and actual values, EchoRCNN has illustrated accurate results.

For the echocardiography sequences chosen for LV segmentation, it is possible to estimate end-diastolic and end-systolic frames. There is a definition for these phases based on the LV volume changes through a cardiac cycle. Therefore, by having the segmented part refer to the LV region in echocardiography sequences, it is possible to estimate the end-diastolic phase to be the frame with maximum LV volume and the end-systolic phase to be the frame with minimum LV volume. However, there is no accurate definition of these two phases in RV functionality in the echocardiography series, so human supervision must be included to help the RV parameter estimation method.

In terms of speed, on only one GPU, the network prediction was 0.08 s per frame, which is considerably fast. We can refer to it as a real-time method that can be used as an assistant and help physicians have more accurate and faster diagnoses.

Our results depict a semi-automated cardiac function evaluation through deep neural networks and can be a significant step in automated cardiac function assessment. The advantage of the proposed method over the same studies in this area is its most minor dependency on the user. The user has to delineate just the first frame of the sequence, whether it is an end-diastolic/end-systolic frame or not. The insufficient dataset of RV is the conspicuous limitation in our work. However, despite using this small dataset, the method has achieved good performance in the RV segmentation task. The main focus of this article is the segmentation of the LV in the echocardiography series. Because of the RV videos amount deficiency, we have used the weights of the trained EchoRCNN on the LV dataset as initial weights for the RV network; hence, we attained reasonable results. In the future, by increasing the datasets and improving the network structure by further researching, the method can predict better.

## Conclusion

We presented EchoRCNN, a deep neural network architecture for echocardiography object segmentation. It needs just the first frame manual segmentation as a guide. Using the segmented part results from passing the sequences through the network and performing the post-processing procedures on the predicted mask led us to have main parameters extracted in echocardiography screening, such as EF from the LV functionality and the FAC from the RV functionality. This automation process speed made us remark the method as a real-time assistant. EchoRCNN is validated on two datasets for evaluating the strategy on LV and RV functionalities. The results indicate a promising performance in the segmentation qualification and the cardiac parameters' estimation. Future work will focus on validating the method on other chambers in the echocardiography series and using a unique network to evaluate all chambers simultaneously.

## Data Availability

Not applicable.

## Appendix

For the first part of the evaluation, Dice Similarity Coefficient (DSC), Jaccard Similarity Coefficient (JSC), precision, and recall metrics are used to evaluate the relativeness of the segmented parts in each frame.

The first evaluation metric is DSC introduced in [36] and is valuable for ecological community data. Given two subsets $X$ and $Y$, DSC is defined as Eq. (1).

1 $${\text{DSC}} = \frac{2|X \cap Y|}{{|X| + |Y|}}$$

JSC is another metric that is not very different from DSC in form and is first introduced in [37]. The main difference is that JSC satisfies the triangle inequality as a proper distance. It is also known as Intersection-over-Union (IoU). The form of this metric for two subsets $X$ and $Y$ is demonstrated in Eq. (2).

2 $${\text{JSC}} = \frac{|X \cap Y|}{{|X \cup Y|}}$$

Precision is another helpful metric in the object segmentation task, also called the positive predicted values. It is defined as the fraction of relative instances among the whole set [38]. Equation (3) defines precision for the $X$ and $Y$ sets, where TP is the true positive value and FP is the false positive value.

3 $${\text{Precision}} = \frac{{{\text{TP}}}}{{{\text{TP}} + {\text{FP}}}}$$

The recall metric is in the context of precision and is also called sensitivity. It is defined as the fraction of the number of relevant pixels retrieved [38]. Equation (4) indicated the recall in a mathematical view where the FN denotes the false negative value.

4 $${\text{Recall}} = \frac{{{\text{TP}}}}{{{\text{TP}} + {\text{FN}}}}$$

For the second part evaluation, which analyzes the extracted echocardiography parameters' accuracy, regression metrics seem suitable because the parameters are continuous variables predicted after the post-processing algorithms on the segmented part. Root Mean Squared Error (RMSE), Mean Absolute Error (MAE), the Coefficient of Determination or R-squared value, and the Cronbach's α are the most popular regression metrics used here.

The RMSE is defined as the root of the mean of the squared errors over the data instances [39], and the MAE is the absolute value of the errors over the whole dataset [40]. Equations (5) and (6) describe the RMSE and MAE formulas. $\widehat{y}$ is the expected value.

5 $${\text{RMSE}} = \sqrt {\frac{1}{n}\sum\limits_{i - 1}^{n} {(y_{i} - \hat{y}_{i} )^{2} } }$$

6 $${\text{MAE}} = \frac{1}{n}\sum\limits_{i = 1}^{n} {|y_{i} - \hat{y}_{i} |}$$

The R-squared is also defined as the ratio of the explained variation to total variations [41]. Equation (7) indicates the R-squared formula. $\widehat{y}$ is the expected value and $\overline{y }$ is the average value of $y$.

7 $$R^{2} = 1 - \frac{{\sum\nolimits_{i = 1}^{n} {(y_{i} - \hat{y}_{i} )^{2} } }}{{\sum\nolimits_{i = 1}^{n} {(y_{i} - \overline{y}_{i} )^{2} } }}\frac{1}{2}$$

The Cronbach's α is a reliability metric; it assumes that all test data points are equivalent test batches and relate the whole test set features by extending one batch's features [42]. Equation (8) indicates the formula of this metric where ${\sigma }_{i}$ is the covariance between the prediction and ground truth and ${\sigma }_{x}$ is the sum of the prediction and ground truth covariance separately.

8 $${\text{Cronbach's}}\;\alpha = \frac{n}{n - 1}\left( {1 - \frac{{\sum\nolimits_{i = 1}^{n} {\sigma_{i}^{2} } }}{{\sigma_{x}^{2} }}} \right)$$

## Abbreviations

a2DQ: Auto 2D Quantification
DAVIS: Densely annotated video object segmentation
DBN: Deep belief network
DSC: Dice similarity coefficient
ECG: Electrocardiogram
EF: Ejection fraction
FAC: Fractional area change
FPN: Feature pyramid network
IoU: Intersection-over-Union
JSC: Jaccard similarity coefficient
LV: Left ventricle
MAE: Mean absolute error
Mask R-CNN: Mask region-based convolutional neural network
OSVOS: One-shot video object segmentation
RGMP: Reference-guided mask propagation
RMSE: Root mean squared error
RV: Right ventricle
SGD: Stochastic gradient descent
SIR: Sampling importance resampling
